# NMN protects cisplatin-associated AKI via NAD^+^/SIRT1 pathway

**DOI:** 10.3389/fimmu.2026.1721884

**Published:** 2026-02-04

**Authors:** Zhaozhi Wen, Jiazeng Wang, Yihang Yang, Longlong Chen, Xiangge Ji, Dong Liu, Chongxu Shi

**Affiliations:** 1 School of Life Sciences, Nantong Laboratory of Development and Diseases, Nantong University, Nantong, Jiangsu, China; 2 Department of Pathology, Affiliated Hospital of Nantong University, Nantong, Jiangsu, China

**Keywords:** acute kidney injury, cisplatin, inflammation, nicotinamide adenine dinucleotide, SIRT1

## Abstract

Acute kidney injury (AKI) is a critical public health concern with high morbidity and mortality. The chemotherapy agent cisplatin is widely used for various solid tumors; however, cisplatin-associated AKI (CIS-AKI) is a frequent complication in the clinic. Nicotinamide adenine dinucleotide (NAD^+^) is a coenzyme central to metabolism and redox reactions. β-Nicotinamide mononucleotide (NMN), a key precursor of NAD^+^, has shown protective effects in various disease models, but its role in CIS-AKI remains unclear. In this study, male mice subjected to CIS-AKI and cisplatin-treated HK-2 cells were employed as *in vivo* and *in vitro* models, respectively, to evaluate the renoprotective effects of NMN. Bulk RNA sequencing revealed marked inflammatory activation and disruption of NAD^+^ metabolism in cisplatin-treated mouse kidneys. NMN administration significantly ameliorated kidney dysfunction, as indicated by reduced plasma creatinine and blood urea nitrogen (BUN) levels, attenuated tubular injury, and decreased expression of kidney injury markers NGAL and KIM-1. It also markedly suppressed kidney inflammation, characterized by reduced IL-6 and IL-18 levels, diminished neutrophil infiltration and macrophage accumulation. Consistently, *in vitro*, NMN attenuated cisplatin-induced reactive oxygen species (ROS) generation and lactate dehydrogenase (LDH) release in HK-2 cells. Mechanistically, NMN elevated kidney NAD^+^ levels and enhanced SIRT1 expression. These findings demonstrate that NMN protects against CIS-AKI by activating the NAD^+^–SIRT1 pathway, thereby reducing oxidative stress and inflammation, and suggest its potential as a therapeutic strategy for CIS-AKI.

## Introduction

Acute kidney injury (AKI) is a severe clinical syndrome characterized by a rapid loss of kidney function, indicated by an increase in plasma creatinine of at least 0.3 mg/dL within 48 hours, or a 1.5-fold rise from baseline within 7 days, or a urine output of no more than 0.5 mL/kg/h for 6 hours or longer (1). With the aging population and rising prevalence of comorbidities such as diabetes, cardiovascular disease, and sepsis, the incidence of AKI continues to increase (2). Despite extensive research, effective therapeutic strategies for AKI remain limited, and mortality rates can reach up to 50% (3). Although supportive care like dialysis can manage the acute phase, the major clinical challenge is preventing long-term kidney function decline following an episode of AKI. This challenge is exemplified by cisplatin, a widely used chemotherapeutic agent whose dose-limiting nephrotoxicity often leads to AKI (4). The underlying mechanisms include mitochondrial dysfunction, oxidative stress, and inflammatory cascades (5). However, the precise signaling networks and their hierarchical roles remain unclear. Sirtuin 1 (SIRT1), a nicotinamide adenine dinucleotide (NAD^+^)-dependent deacetylase, plays a crucial role in regulating cellular metabolism, aging, apoptosis, inflammation, and oxidative stress (6, 7). Previous studies have shown that SIRT1 activation offers protection against AKI through multiple pathways, including SIRT1/Nrf2/HO-1 and SIRT1/NF-κB (8). Nicotinamide mononucleotide (NMN), a direct precursor of NAD^+^, activates SIRT1-dependent pathways via restoring cellular NAD^+^ levels (9). In preclinical studies, NMN has protective effects on aging, neurodegeneration, and metabolic disorders (10). However, the role of NAD^+^-SIRT1 axis in CIS-AKI remains unclear. Therefore, we hypothesized that NMN can alleviate CIS-AKI, tubular injury, inflammatory response, and ROS release by upregulating NAD^+^ levels and activating SIRT1.

## Methods

### Animal study

Male C57/BL6J mice (20–25 g) were purchased from the Animal Experimental Center of Nantong University and maintained under standard conditions (22 ± 2°C, 50–60% humidity, 12 h light/dark cycle) with unlimited access to food and water. Mice were randomly assigned to four groups (n = 6 per group): Control (0.9% saline, i.p.); Cisplatin (23 mg/kg, i.p.); Cisplatin + NMN-low (500 mg/kg, i.p.); Cisplatin + NMN-high (1000 mg/kg, i.p.). Cisplatin (Qilu Pharmaceutical, China) was dissolved in 0.9% saline. NMN (β-nicotinamide mononucleotide; Beyotime, China) was dissolved in 0.9% saline and administered intraperitoneally (i.p.) once daily for four consecutive days. The second dose of NMN was administered 1 h before cisplatin injection. For blood collection under anesthesia, mice received a single intraperitoneal injection of 2,2,2-tribromoethanol (250 mg/kg, 2.5% w/v in saline, 10 ml/kg body weight; Shanghai Yuanye Bio-Technology, China). Mice were euthanized 72 h post-cisplatin injection; blood and kidneys were harvested for analysis. Euthanasia was performed by cervical dislocation by trained personnel after confirming loss of consciousness. All animal procedures were approved by the Institutional Animal Care and Use Committee of Nantong University and conducted under the NIH Guide for the Care and Use of Laboratory Animals.

### *In vitro* study

HK-2 cells (human kidney-2; ATCC CRL-2190) were obtained from Bluefbio Co. (China) and cultured in Dulbecco’s Modified Eagle Medium (DMEM; Pricella, China) supplemented with 10% fetal bovine serum (FBS; Bio-Channel, China) and 1% penicillin–streptomycin (PS, Beyotime, China) at 37°C in a humidified atmosphere of 5% CO_2_. For lactate dehydrogenase (LDH) assays, HK-2 cells were seeded into 96-well plates (3 × 10^5^ cells/well) and cultured for 24 h until 80% confluence. For RNA extraction, HK-2 cells were seeded into six-well plates (1 × 10^6^ cells/well) and cultured for 24 h until 80% confluence. To detect reactive oxygen species (ROS) release, HK-2 cells were seeded onto sterile glass coverslips and placed in 24-well plates (2 × 10^5^ cells/well) and cultured for 24 h until 80% confluence. For SIRT1 inhibition experiments, 10 μM EX-527 (Beyotime, China) was applied simultaneously with NMN for 24 h.

### RNA-sequencing and bioinformatics analysis

Fresh kidney tissues were snap-frozen in liquid nitrogen and shipped on dry ice to Singleron Biotechnologies (China) within 2 h. Total RNA was extracted using TRIzol reagent and subjected to library construction with the GEXSCOPE^®^ Universal RNA Library Kit. Paired-end 150-bp sequencing was performed on an Illumina NovaSeq 6000. Clean reads were aligned to the mouse reference genome (GRCm38) with HISAT2. Differential expression (|log_2_FC| ≥ 1, adjusted P < 0.05) and pathway enrichment were analyzed using DESeq2 (version 1.32.0) and clusterProfiler (version 4.2.0), respectively. All analyses were performed using R (version 4.1.2).

### Kidney function assessment

To evaluate kidney function, blood urea nitrogen (BUN) and plasma creatinine levels were measured by a urea assay kit and a creatinine assay kit (Maryland, USA), respectively.

### Histological assessment

Fresh kidneys were fixed in 4% paraformaldehyde for 24 h, and then embedded in paraffin. Sections (4 µm) were stained with hematoxylin and eosin (H&E) and examined at ×100 or ×200 magnification. Twenty random fields per section (×200) were graded on a 0–5 scale: 0 (<10%), 1 (10–30%), 2 (30–50%), 3 (50–70%), 4 (70–90%), or 5 (>90%) of tubules showing dilation, brush-border loss, casts, or necrosis.

### Immunohistochemistry

Immunohistochemical staining was performed by Friss Biotechnology (China) using the following primary antibodies: anti-SIRT1 (1:250, catalog number 60303-1-Ig; Proteintech, China), anti-Ly6G (1:200, catalog number 24633-1-AP; Proteintech, China), anti-NGAL (1:1000, catalog number ab216462; Abcam, UK), anti-F4/80 (1:2000, catalog number 28463-1-AP; Proteintech, China), and anti-KIM-1 (1:200, catalog number PA5-98302; Proteintech, China). Light microscopy was employed for image acquisition, followed by semi-quantitative analysis of protein expression using ImageJ software.

### Quantitative real-time PCR analysis

Total RNA from kidneys was isolated using a Total RNA Extraction Kit (CWBIO, China) according to the manufacturer’s experimental protocols. Then, the concentration of RNA was measured using a microvolume spectrophotometer (Thermo Fisher Scientific, Waltham, USA). After reverse transcription, quantitative real-time PCR was performed using the Fast qPCR Kit (Kapa Biosystems, USA) on a PCR system (CFX Connect; Bio-Rad, USA). Primer sequences are listed in **Supplementary Table 1**. Relative gene expression was calculated using the 2^−ΔΔCt method.

### Biochemical assays

The levels of LDH, ROS, and NAD^+^ in tissue lysates and HK-2 cells were quantified using the LDH assay kit (Beyotime, China), the ROS assay kit (Beyotime, China), and the NAD^+^/NADH assay kit (Beyotime, China), respectively, and strictly adhering to the manufacturers’ prescribed protocols.

### Statistical analysis

Statistical analysis was performed using one-way ANOVA to assess differences among the groups. Following the ANOVA, pairwise comparisons between the groups were conducted using *post-hoc* tests to identify significant differences. Data are presented as mean ± SEM, and a P-value < 0.05 was considered statistically significant.

## Result

### Bulk RNA-seq profiling reveals cisplatin-induced inflammatory response and disrupted NAD^+^ metabolism in the mouse kidney

**Figure 1:**
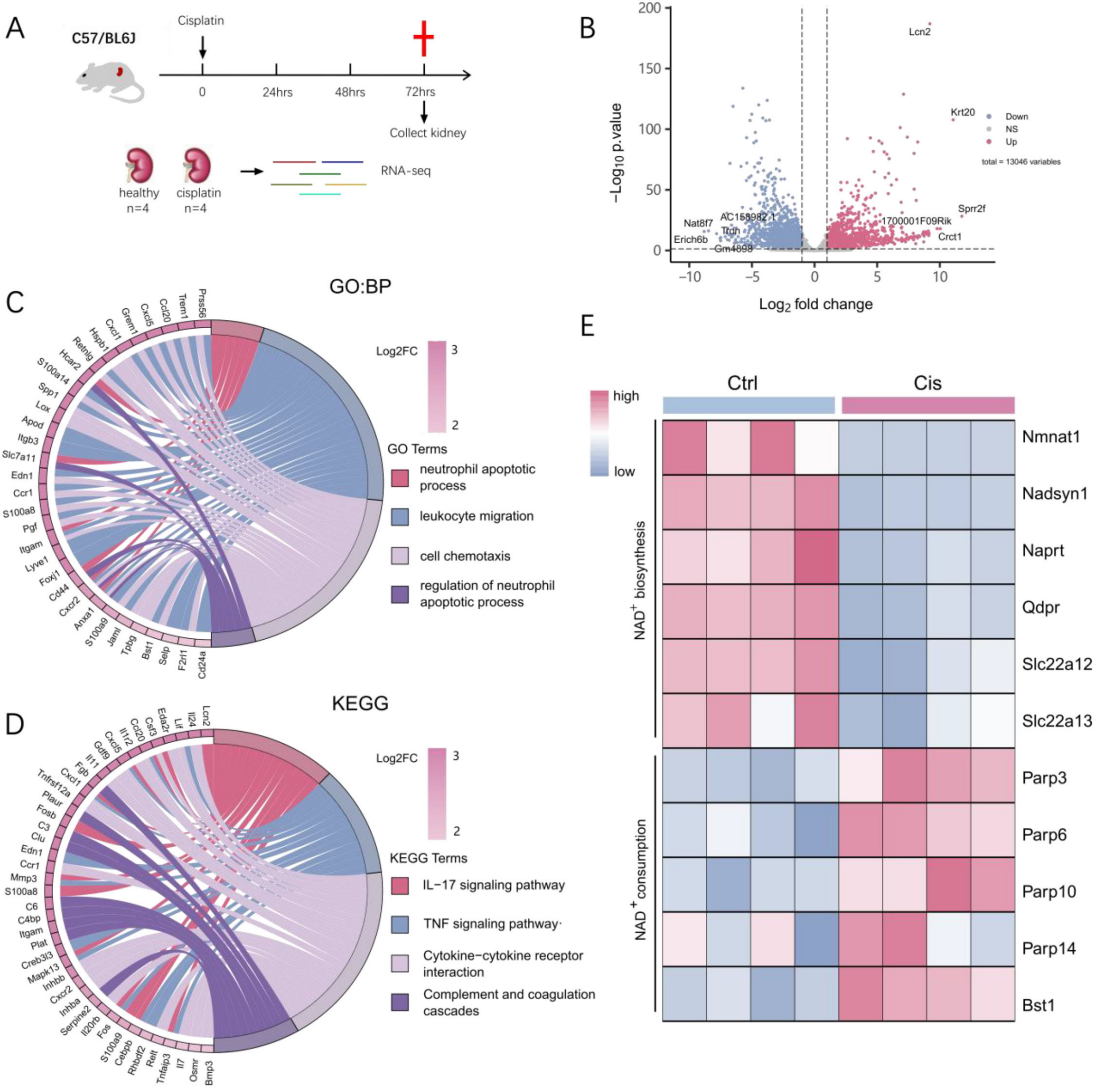
Transcriptomic analysis of CIS-AKI in mice. **(A)** Experimental timeline and workflow. Mice were randomized into two groups (n = 4 per group): the control group and the cisplatin group. Kidneys were harvested for RNA sequencing at 72 h post-injection. **(B)** Volcano plot showed DEGs between cisplatin-treated and control kidneys. X-axis: log_2_ (fold change); y-axis: –log_10_ (adjusted P-value). Significance thresholds: |log_2_ FC| ≥ 1 and adjusted P-value < 0.05. The top five up- and down-regulated genes are labeled. **(C)** GO chord plot showed the relationship between selected genes and their GO terms along with gene log_2_FC. The selected four GO terms were chosen from the top ten most significant terms for visualization. The left half displayed up-regulated DEGs, while the right half represents different GO terms in varied colors. Genes were linked to GO terms by colored bands. **(D)** KEGG chord plot showed the relationship between selected genes and their KEGG terms along with gene log_2_FC. The selected four KEGG terms were chosen from the top ten most significant terms for visualization. The left half displayed up-regulated DEGs, while the right half represented different KEGG terms in varied colors. Genes were linked to KEGG terms by colored bands. **(E)** Heatmap showing NAD^+^ synthesis and consumption-related genes across all samples. The color gradient represents the expression levels from low (blue) to high (red).

To characterize the molecular landscape of CIS-AKI, we performed bulk RNA-seq on kidneys (**Figure 1A**). Differential expression gene (DEG) analysis identified 2,387 up-regulated and 4,774 down-regulated genes (|log_2_ FC| ≥ 1, adjusted P-value < 0.05) (**Figure 1B**; **Supplementary Table 2**). The top up-regulated genes included *Lcn2*, *Havcr1*, *Il1f6*, *Il24*, *Saa1* and *Timp1*. All those genes are implicated in inflammatory signaling and immune cell recruitment (**Figure 1D**).

To better understand the biological significance of the up-regulated genes identified in our study, we employed Gene Ontology (GO) and Kyoto Encyclopedia of Genes and Genomes (KEGG) analyses. The results were visualized using chord plots to provide an intuitive representation of the enriched biological processes and pathways. GO enrichment of the up-regulated genes revealed significant enrichment of biological processes such as “neutrophil apoptotic process”, “leukocyte migration”, and “cell chemotaxis” (**Figure 1C**; **Supplementary Table 3**). KEGG pathway analysis further mapped these genes to the IL-17 signaling, TNF signaling, and cytokine–cytokine receptor interaction (**Figure 1D**; **Supplementary Table 4**). Both the GO and KEGG chord plots visualized these relationships, highlighting a pronounced pro-inflammatory response. Gene Set Enrichment Analysis (GSEA) further confirmed activation of IL-17 and TNF signaling alongside p53-mediated stress response, indicating concurrent inflammatory and pro-apoptotic programs in cisplatin-injured kidneys (**Supplementary Figure S1**; **Supplementary Table 5**).

Because NAD^+^ availability is critical for kidney stress adaptation (11), we next analyzed the expression of genes regulating NAD^+^ biosynthesis and consumption. Compared with controls, kidneys from cisplatin-treated mice exhibited marked down-regulation of biosynthetic enzymes (*Nmnat1*, *Nadsyn1*, *Naprt*, *Qdpr*, *Slc22a12*, *Slc22a12*) and accompanying up-regulation of NAD^+^ consumers (*Parp3*, *Parp6*, *Parp10*, *Parp14*, *Bst1*) (**Figure 1E**). These reciprocal changes indicate a severe disruption of NAD^+^ homeostasis during CIS-AKI. Additionally, we validated the expression of the aforementioned genes involved in NAD^+^ biosynthesis and consumption pathways using quantitative real-time PCR (qPCR). The qPCR results corroborated the RNA-seq data, showing consistent up-regulation or down-regulation of these genes (**Supplementary Figure S2**).

### NMN preserves cisplatin-induced kidney function loss

**Figure 2:**
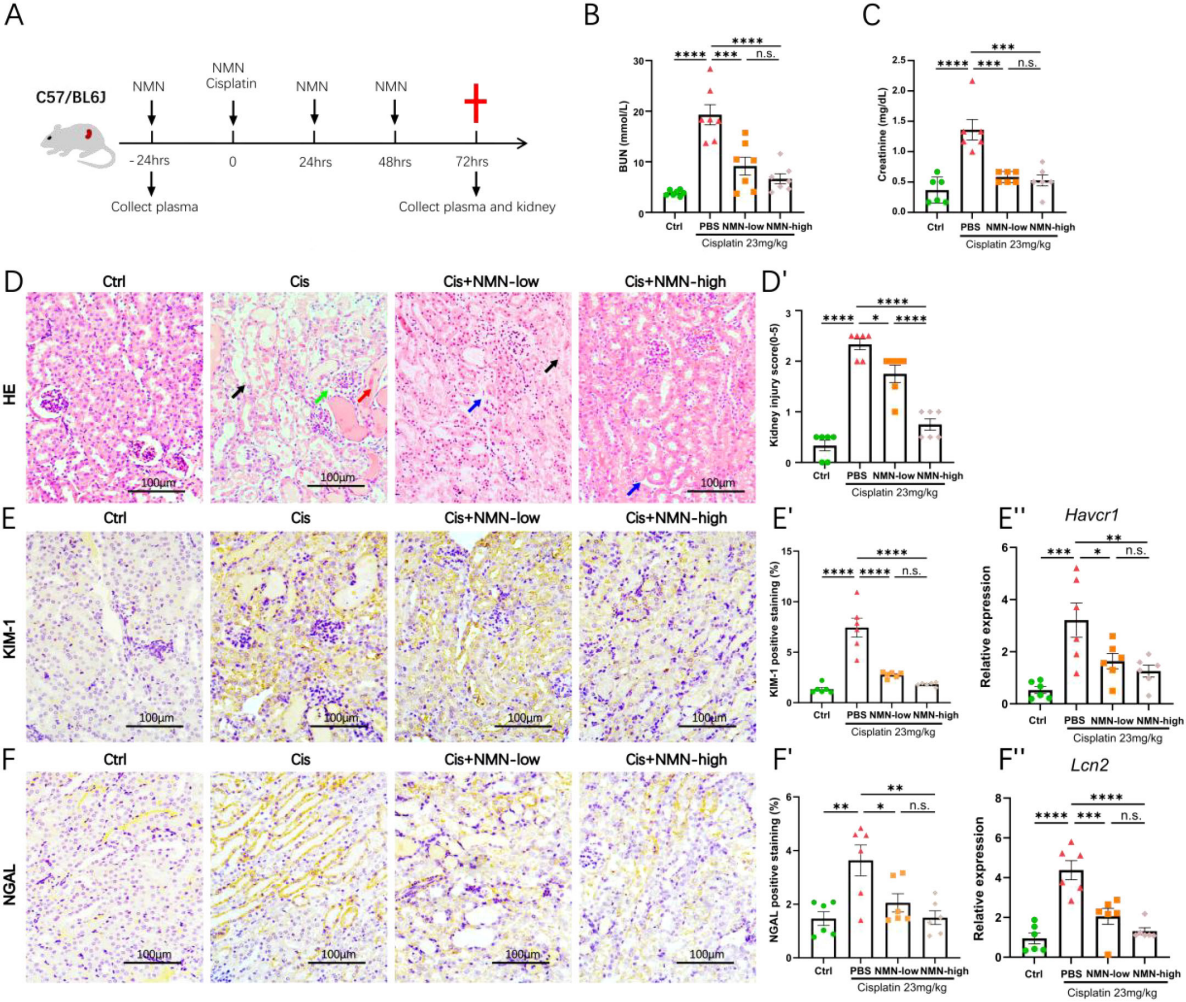
NMN attenuates cisplatin-induced kidney injury. Mice were randomly divided into four groups (n=6 per group; plasma blood urea nitrogen (BUN) n=7): the control group, the cisplatin group, the cisplatin with 500 mg/kg NMN group (labeled as NMN-low in the figure), and the cisplatin with 1000 mg/kg NMN group (labeled as NMN-high in the figure). **(A)** Experiment scheme. **(B, C)** Quantification of plasma BUN and creatinine levels in four groups of mice. **(D)** Representative image of HE stained mouse kidney from four groups. The arrows in the images specifically point to areas of pathological damage: black to signs of tubular necrosis, red to the presence of tubular casts, blue to loss of the brush border, and green to dilation of the proximal tubules. **(D’)** Quantification of kidney injury scores. **(E)** Representative images of KIM-1-stained mouse kidney. **(E’)** Quantification of KIM-1-positive staining area. **(E’’)** qPCR analysis of *Havcr1* mRNA levels. **(F)** Representative images of NGAL-stained mouse kidney. **F’.** Quantification of NGAL-positive staining area. **(F’’)** qPCR analysis of *Lcn2* mRNA levels. Quantification of positive staining area is expressed as percentage of total field (positive area/total area × 100%) measured by ImageJ with color-threshold segmentation. qPCR data are normalized to *18s* and shown as fold-change versus control. Data were presented as mean ± SEM. Statistical significance was determined using one-way ANOVA followed by Tukey’s *post hoc* test. *p < 0.05, **p < 0.01, ***p < 0.001, and ****p < 0.0001 indicate significant differences between groups. Scale bars as 100 µm.

To comprehensively evaluate the renoprotective efficacy of NMN against CIS-AKI, mice were subjected to a preventive schedule (**Figure 2A**). Functional assessment revealed that NMN significantly attenuated cisplatin-induced plasma creatinine and BUN increase in a dose-dependent manner, indicating NMN improved glomerular filtration capacity and overall kidney performance (**Figures 2B, C**). Histopathological examination of H&E demonstrated that cisplatin-induced extensive proximal tubular dilation, marked loss of brush border integrity, intraluminal cast formation, and focal tubular necrosis (**Figure 2D**). And these structural abnormalities were strikingly ameliorated by NMN pretreatment, with the high dose exerting the most pronounced protection, as evidenced by a significant reduction in tubular injury scores (**Figure 2D**).

Immunohistochemical staining showed that cisplatin triggered robust cortical expression of neutrophil gelatinase-associated lipocalin (NGAL, encoded by the *Lcn2* gene) and kidney injury molecule-1 (KIM-1, encoded by the *Havcr1* gene), predominantly in the dilated proximal tubules (**Figures 2E, F**). NMN pretreatment dose-dependently reduced both signals, with the high-dose group approaching baseline levels, corroborating the histological protection observed in **Figure 2D**.

Consistently, cortical qPCR revealed the same trend: cisplatin-induced up-regulation of *Lcn2* and *Havcr1* mRNA was dose-dependently reversed by NMN (**Figures 2E’’, F’’**).

Together, these data demonstrate that prophylactic NMN preserves kidney function and limits tubular injury in CIS-AKI.

### NMN suppresses cisplatin-induced inflammatory response

**Figure 3:**
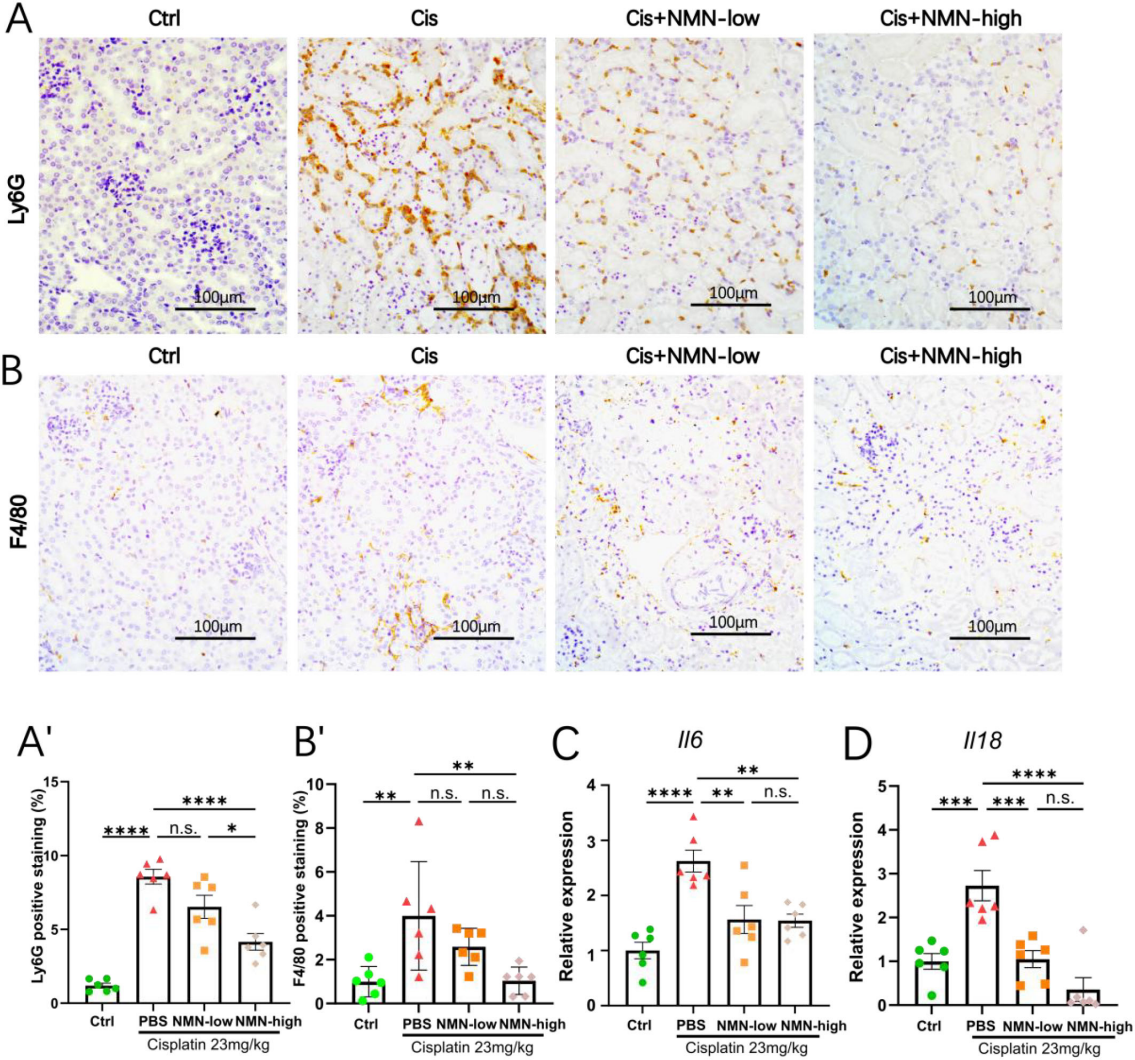
NMN suppresses cisplatin-induced renal inflammation. **(A)** Representative images of Ly6G-stained kidney tissues. **(A’)** Quantification of Ly6G-positive staining area. **(B)** Representative images of F4/80-stained kidney tissues. **(B’)** Quantification of F4/80-positive staining area. **(C, D)** qPCR analysis of *Il6* and *Il18* mRNA levels. Quantification of positive staining area is expressed as percentage of total field (positive area/total area ×100%) measured by ImageJ with color-threshold segmentation. qPCR data are normalized to *18s* and shown as fold-change versus control. Data are mean ± SEM; statistical significance was determined using one-way ANOVA followed by Tukey’s *post hoc* test. *p < 0.05, **p < 0.01, ***p < 0.001, and ****p < 0.0001 indicate significant differences between groups.

Inflammatory cell infiltration, particularly neutrophil recruitment, is not only a marker but also a driver of tissue damage, amplifying oxidative stress and tubular injury (12). To assess the inflammatory response in CIS-AKI, we employed immunohistochemistry with anti-Ly6G antibody to label neutrophil infiltration. Cisplatin-treated kidneys exhibited prominent accumulation of Ly6G-positive neutrophils in both cortical and medullary regions, whereas 1000 mg/kg NMN treatment markedly reduced neutrophil infiltration (**Figure 3A**). Consistently, F4/80 immunostaining revealed a robust accumulation of macrophages in the corticomedullary junction and peritubular areas of cisplatin-injured kidneys, which was likewise attenuated by NMN (**Figure 3B**). Additionally, qPCR analysis demonstrated that NMN effectively attenuated pro-inflammatory cytokine gene expression, indicating NMN has potent anti-inflammatory activity (**Figures 3C, D**). Thus, these results indicated that NMN reduced kidney inflammation in CIS-AKI by limiting neutrophil infiltration, macrophage recruitment, and pro-inflammatory cytokine expression.

### NMN restores NAD^+^ balance and upregulates SIRT1 expression in cisplatin-kidney

Our bulk RNA-seq analysis found that cisplatin-induced severe disruption of NAD^+^ homeostasis in the mouse kidney; therefore, we hypothesized that replenishing the NAD^+^ pool with its precursor NMN could restore kidney metabolic homeostasis and protect from CIS-AKI. SIRT1 is an NAD^+^-dependent deacetylase that governs oxidative stress, inflammation and cell survival, all of which drive cisplatin nephrotoxicity. Prior work showed SIRT1 activity declines in injured kidneys and that its pharmacological activation is protective (8). We therefore asked whether NMN restores NAD^+^ availability and subsequently enhances *Sirt1* expression in cisplatin-treated kidneys.

**Figure 4:**
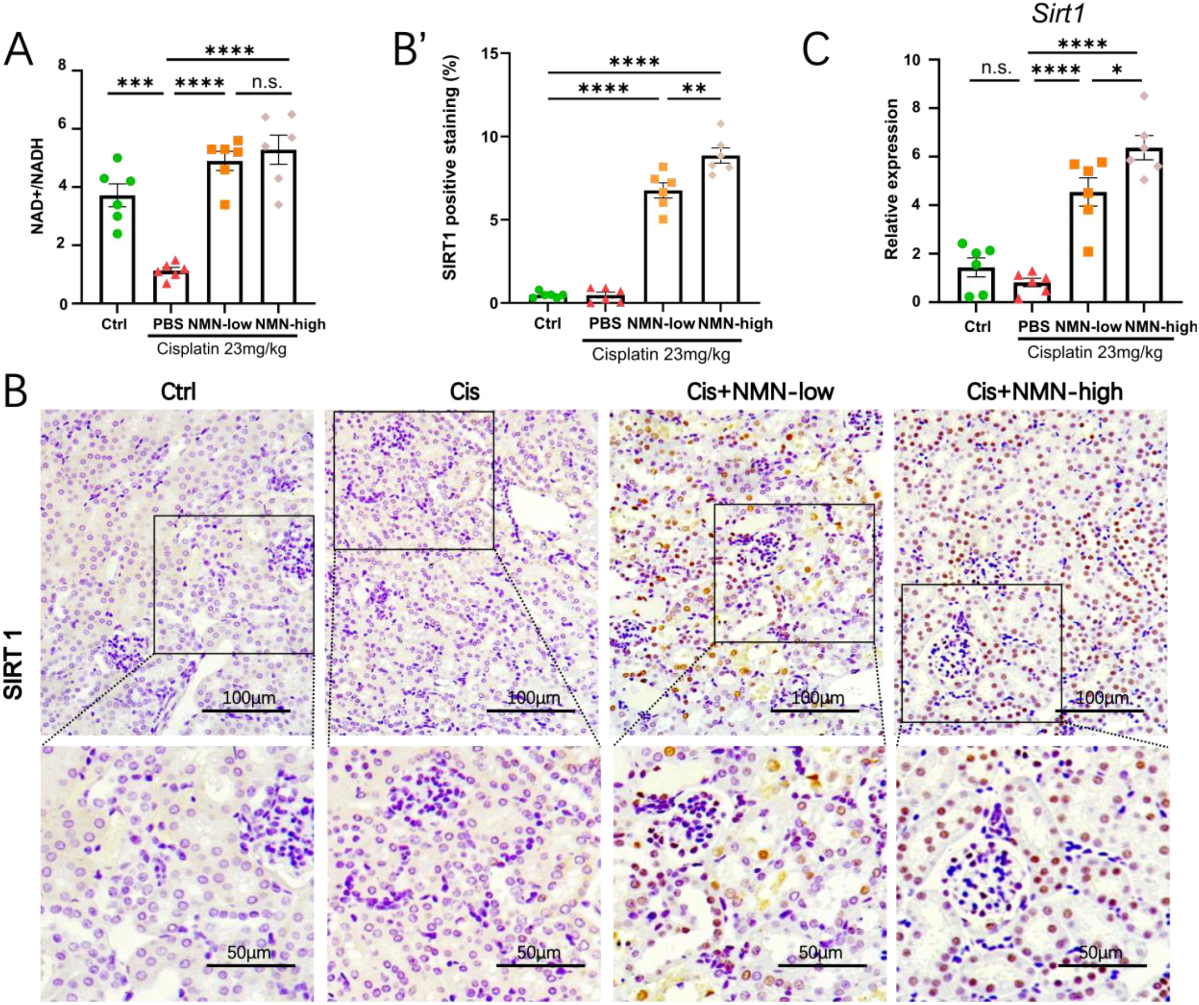
NMN restored NAD^+^ balance and upregulated SIRT1 expression in kidney tissue. **(A)** Measurement of the NAD^+^/NADH ratio. **(B)** Representative images of SIRT1-stained kidney tissues; lower panels show magnified views (scale bar = 50 µm). **(B’)** Quantification of SIRT1-positive staining area. **(C)** qPCR analysis of *Sirt1* mRNA levels. Quantification of positive staining area is expressed as percentage of total field (positive area/total area × 100%) measured by ImageJ with color-threshold segmentation. qPCR data are normalized to *18s* and shown as fold-change versus control. Data are mean ± SEM; statistical significance was determined using one-way ANOVA followed by Tukey’s *post hoc* test. *p < 0.05, **p < 0.01, ***p < 0.001 and ****p < 0.0001 indicate significant differences compared to the control group. Scale bars as100 µm.

Firstly, kidney NAD^+^/NADH ratios were analyzed. NMN administration elevated NAD^+^/NADH ratio in a dose-dependent manner, indicating NMN effectively restored kidney NAD^+^ levels (**Figure 4A**). Furthermore, we observed SIRT1 protein signals in tubular epithelial cells, displaying faint, diffuse cytoplasmic and nuclear signals in the control group. And cisplatin did not increase SIRT1 levels in the kidney. Maintaining similar faint, diffuse staining patterns. Interestingly, NMN treatment markedly intensified SIRT1 levels, with pronounced nuclear localization evident as strong brown granules, suggesting enhanced enzymatic activation (**Figure 4B**). qPCR further confirmed that *Sirt1* mRNA levels rose in parallel with increasing NMN dose. (**Figure 4C**).

Collectively, these findings indicated that NMN rapidly rebuilds kidney NAD^+^ reserves and concomitantly up-regulates SIRT1 expression, thereby activating the NAD^+^–SIRT1 axis. This metabolic–epigenetic crosstalk mediates the renoprotective effects of NMN against cisplatin-induced nephrotoxicity.

### NMN protects against cisplatin-induced tubular epithelial cell injury

**Figure 5:**
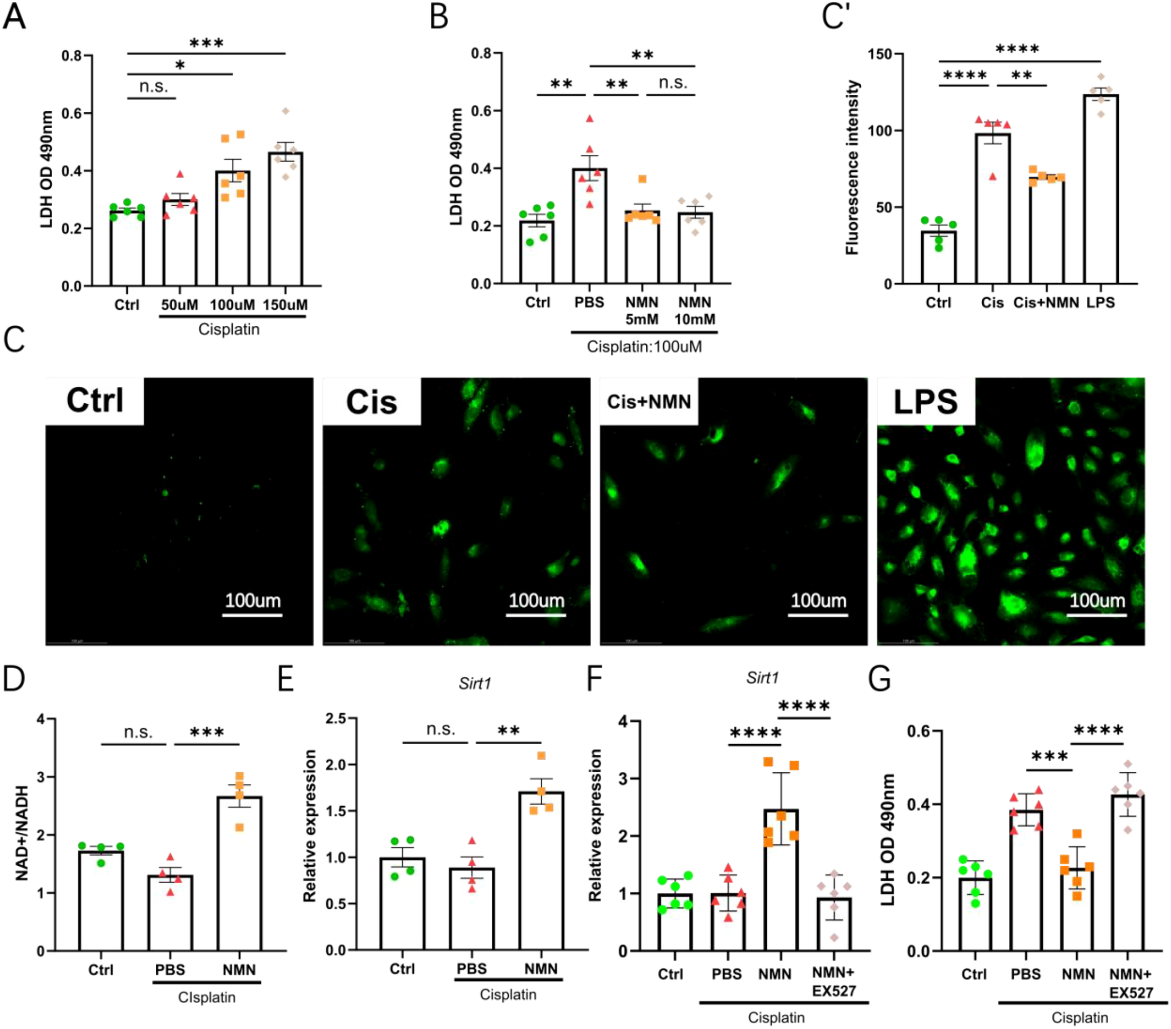
NMN protects HK-2 cells against cisplatin-induced injury in a SIRT1-dependent manner. **(A, B)** Lactate dehydrogenase (LDH)-release assays screening optimal doses of cisplatin **(A)** and NMN **(B)** in HK-2 cells (24 h). **(C)** Representative ROS fluorescence images. **(C’)** Quantification of ROS intensity. **(D)** NAD^+^/NADH ratio. **(E)** qPCR analysis of *Sirt1* gene expression. **(F)** qPCR analysis of *Sirt1* mRNA levels in the four experimental groups (Control, Cis, Cis+NMN, Cis+NMN+EX-527). **(G)** LDH release in the four experimental groups. qPCR data are normalized to *β-actin* and shown as fold-change versus control. Data are mean ± SEM; statistical significance was determined using one-way ANOVA followed by Tukey’s *post hoc* test. *p < 0.05, **p < 0.01, ***p < 0.001, ****p < 0.0001 indicate significant differences between groups. Scale bar is 100 µm.

To corroborate our *in vivo* findings, we established an *in vitro* model of cisplatin nephrotoxicity using the human proximal tubular epithelial cell line HK-2. Cells were pre-treated with vehicle or 5 mM NMN for 2 h and then challenged with 100 µM cisplatin for 24 h (concentrations selected from lactate dehydrogenase-release dose–response screening [LDH] as optimal) (**Figures 5A, B**). In parallel, 100 µg/mL LPS (24 h) was employed as a positive control for oxidative stress and produced a robust intracellular ROS burst. Cisplatin evoked a comparable rise in DCFH-DA fluorescence, whereas NMN pre-treatment diminished ROS generation (**Figure 5C**), demonstrating effective cytoprotection and antioxidant activity.

We next examined the intracellular NAD^+^/NADH ratio and *Sirt1* expression. Unexpectedly, cisplatin alone did not significantly alter the NAD^+^/NADH ratio or *Sirt1* mRNA levels in HK-2 cells (**Figures 5D, E**), a finding that contrasts with the clear NAD^+^ depletion observed *in vivo* (**Figure 4**). This discrepancy likely reflects the shorter insult duration, the absence of systemic inflammatory cues. Nevertheless, supplementation with 5 mM NMN elevated the intracellular NAD^+^/NADH ratio and increased *Sirt1* transcript abundance (**Figures 5D, E**), indicating that exogenous NMN is sufficient to activate the NAD^+^/SIRT1 axis even under mild baseline injury.

To determine whether the cytoprotective actions of NMN require SIRT1 activity, we co-incubated cells with the selective SIRT1 inhibitor EX-527 (10 µM) applied simultaneously with NMN. EX-527 completely abolished the NMN-induced increase in *Sirt1* mRNA (**Figure 5F**). This observed reduction in *Sirt1* mRNA following pharmacological inhibition aligns with the broader principle that the activity state of the SIRT1 pathway is intrinsically linked to the transcriptional regulation of its components, as similarly demonstrated by interventions targeting this pathway in other models (13). Consequently, the ability of NMN to reduce cisplatin-stimulated LDH release was fully reversed (**Figure 5G**), establishing that the renoprotective effect of NMN is SIRT1-dependent.

Collectively, these data show that NMN protects tubular epithelial cells against cisplatin-induced injury by restoring NAD^+^ homeostasis, suppressing oxidative stress, and up-regulating the NAD^+^/SIRT1 pathway (**Figure 5F**).

## Discussion

We hypothesized that NAD^+^ precursor, NMN, protects against CIS-AKI by restoring NAD^+^ homeostasis and enhancing SIRT1 expression. Our findings suggested that the NAD^+^–SIRT1 axis plays a central role in the CIS-AKI, implying NMN as a promising renoprotective agent.

As an NAD^+^-dependent deacetylase, SIRT1 orchestrates cellular stress responses by deacetylating key transcriptional regulators, including PGC-1α, FOXO3a, NF-κB, and Nrf2, thereby modulating mitochondrial biogenesis, redox homeostasis, apoptotic signaling, and inflammatory cascades (14). In the present study, NMN-mediated up-regulation of SIRT1 was closely associated with attenuated tubular injury, decreased expression of pro-inflammatory cytokines (IL-6, IL-18), reduced neutrophil infiltration, and diminished oxidative stress, implying that restoration of SIRT1 activity is a critical mechanism underlying the renoprotective effects of NMN in CIS-AKI.

Moreover, NMN not only increased SIRT1 abundance but also altered its subcellular distribution: immunohistochemical analysis revealed that NMN treatment shifted SIRT1 from a diffuse and weak cytoplasmic/nuclear pattern observed in control kidneys to a pronounced nuclear accumulation, indicating enhanced enzymatic activation. This dynamic redistribution is consistent with previous reports demonstrating stress-induced nuclear translocation of SIRT1 during metabolic or oxidative challenges (15), further corroborating that NAD^+^ repletion augments SIRT1 functionality through both quantitative and spatial regulation.

While several studies have explored the protective role of other NAD^+^ precursors such as nicotinamide riboside (NR) and nicotinamide (NAM), the application of NMN in CIS-AKI has not been extensively investigated. Yan Jia et al. (2022) reported that NMN reduces kidney ischemia–reperfusion injury (IRI) through NAD^+^ replenishment and mitochondrial protection, supporting the therapeutic potential of NMN in other AKI models (16). Our studies provided further insight by revealing that the renoprotective effects of NMN are associated with SIRT1 activation and reduced inflammatory response in a CIS-AKI model.

Of note, NMN was administered prophylactically in this study. Although this approach was useful for establishing mechanistic insights, it may not fully reflect clinical practice, where treatment typically begins after injury. Therefore, future studies should evaluate the efficacy of delayed NMN intervention and assess its potential to mitigate long-term consequences such as fibrosis and chronic kidney disease (CKD) (17). Another limitation is that definitive evidence for SIRT1-dependency was obtained only at the cellular level. Although the correlation is strong, definitive causality requires further studies using SIRT1 inhibitors (such as EX-527 (18)) or renal-specific knockout models in mice. Nevertheless, the consistency between transcriptomic data, biochemical assays, and *in vitro* observations strengthens our conclusion that NAD^+^ repletion and SIRT1 activation are central to NMN’s protective mechanism (19). Importantly, NMN co-treatment did not impair cisplatin-mediated tumor suppression (20), supporting its safe adjunct use.

**Figure 6:**
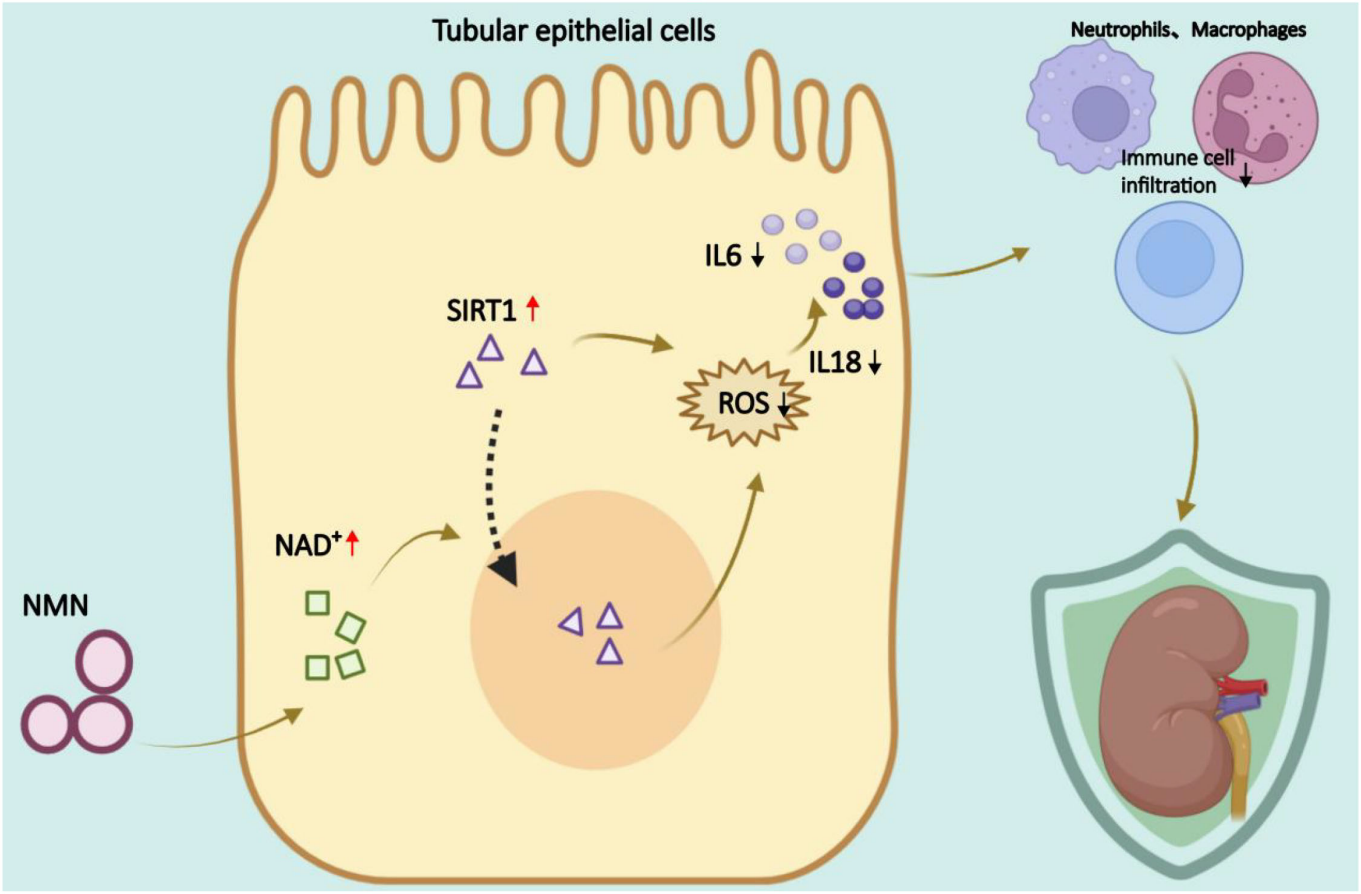
Schematic diagram of NMN-mediated kidney protection in CIS-AKI. NMN restores NAD^+^ levels and subsequently activates SIRT1, thereby diminishing oxidative stress and downstream pro-inflammatory cytokine expression, leading to a marked reduction in immune cell infiltration and overall kidney protection.

In summary, our findings revealed that NAD^+^ depletion and inflammatory activation are prominent features of CIS-AKI. NMN treatment restored NAD^+^ balance, enhanced SIRT1 expression, and alleviated kidney injury. These results provide a strong rationale for further development of NAD^+^-based interventions for AKI (**Figure 6**).

## Data Availability

The original contributions presented in the study are publicly available. This data can be found here: NCBI GEO (accession number GSE306603).
