# Supplementary material for: NMN protects cisplatin-associated AKI via NAD+/SIRT1 pathway
Source: Front Immunol. 2026 Feb 4;17:1721884. doi: 10.3389/fimmu.2026.1721884 (PMC12913174; doi:10.3389/fimmu.2026.1721884)
Supplement: Supplementary file 6 [file DataSheet1.docx]

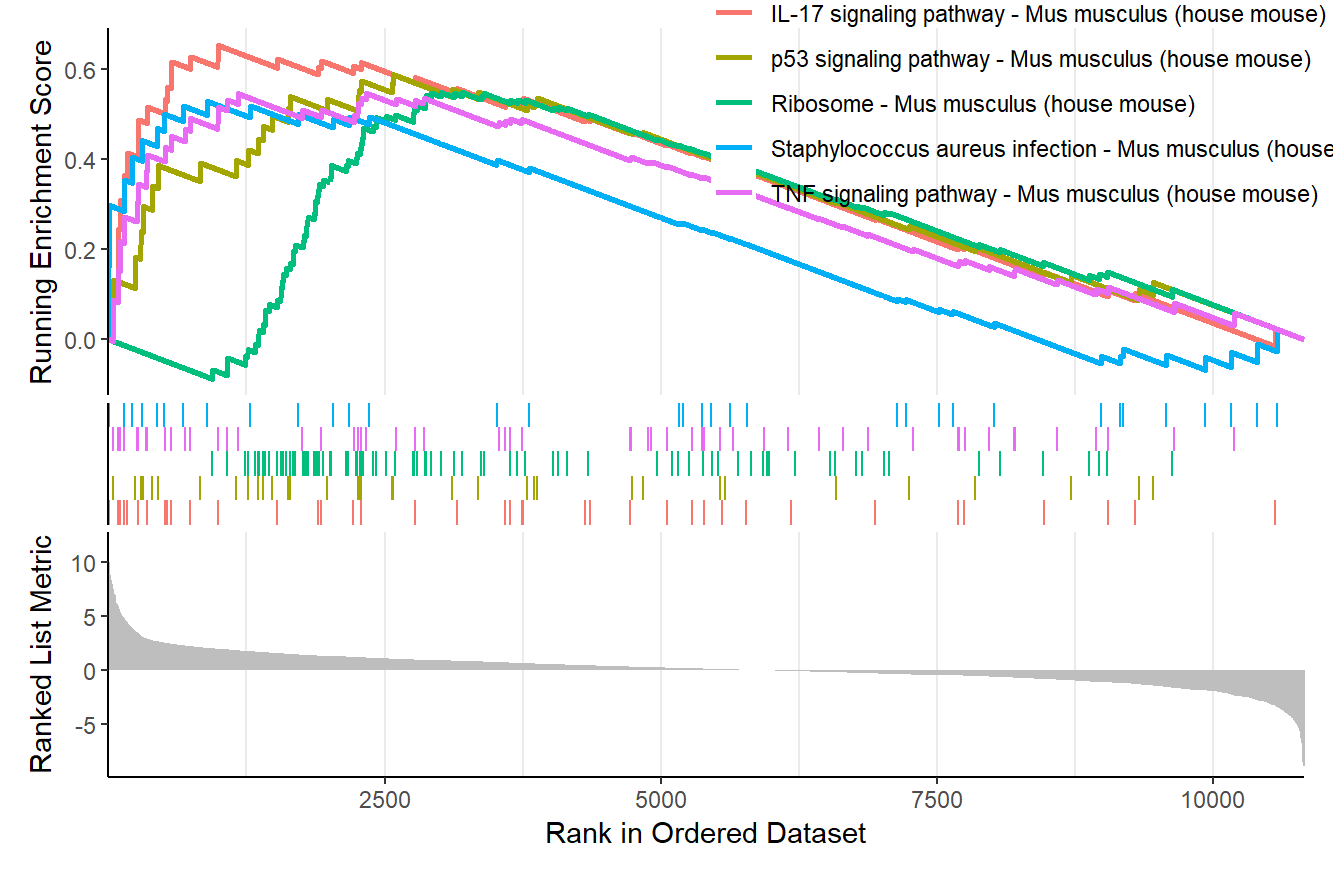


**Figure S1. Gene set enrichment analysis.** GSEA of KEGG gene sets ranked by signal-to-noise metric between cisplatin-treated and control groups. The top five pathways by |NES| are shown. All shown pathways are significant at FDR q < 0.05; exact NES and q values are provided in Table S3.
