# Supplementary material for: NMN protects cisplatin-associated AKI via NAD+/SIRT1 pathway
Source: Front Immunol. 2026 Feb 4;17:1721884. doi: 10.3389/fimmu.2026.1721884 (PMC12913174; doi:10.3389/fimmu.2026.1721884)
Supplement: Supplementary file 7 [file DataSheet2.docx]

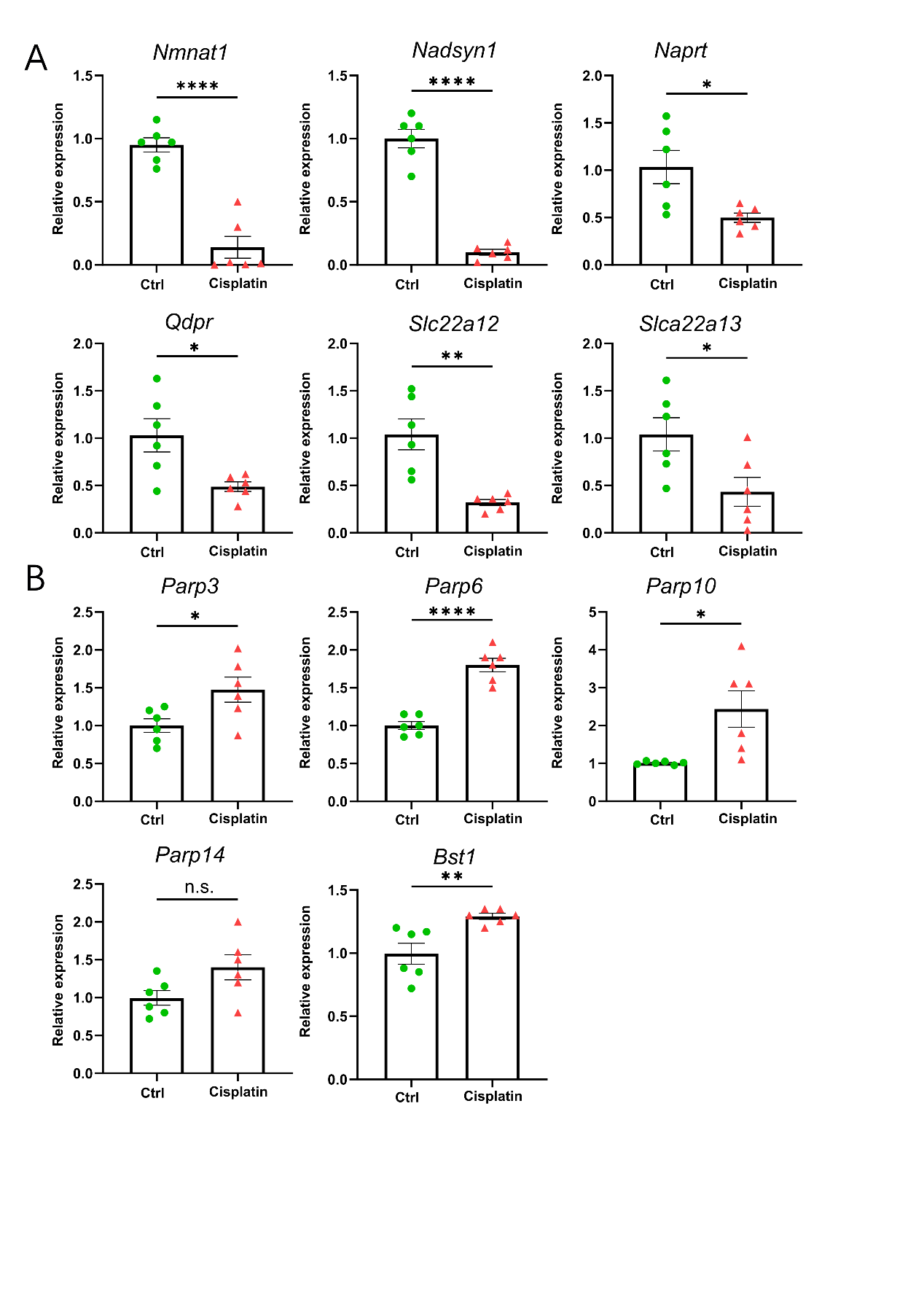


**Figure S2. NAD^+^ related gene expression in control and cisplatin groups.** qPCR analysis of mRNA expression levels for **A.** NAD⁺ synthesis-related genes and **B.** NAD⁺ consumption-related genes in kidney tissue. Gene expression was normalized to the housekeeping gene *18s*. Data were presented as mean ± SEM. Statistical significance was determined using one-way ANOVA followed by Tukey's post hoc test. *p < 0.05, **p < 0.01, ***p < 0.001, and ****p < 0.0001 indicate significant differences between groups.
